# Overexpression of MAGE-A9 Is Predictive of Poor Prognosis in Epithelial Ovarian Cancer

**DOI:** 10.1038/srep12104

**Published:** 2015-07-15

**Authors:** Yunzhao Xu, Chenyi Wang, Yuquan Zhang, Lizhou Jia, Jianfei Huang

**Affiliations:** 1 Department of Obstetrics and Gynecology, Nantong University Affiliated Hospital, Nantong 226001, Jiangsu, China; 2 Department of Obstetrics and Gynecology, The Affiliated People’s Hospital of Inner Mongolia Medical College, Inner Mongolia Autonomous Region 010021, China; 3 Department of Pathology, Nantong University Affiliated Hospital, Nantong 226001, Jiangsu, China

## Abstract

The cancer testis antigen, melanoma-associated antigen A9 (MAGE-A9), is expressed in many kinds of different human cancers, and is an important target for immunotherapy. However, the clinicopathologic significance of MAGE-A9 in epithelial ovarian cancer (EOC) is unknown. In this study, real-time PCR (12 carcinomas of high FIGO stage, 12 carcinomas of low FIGO stage, and 20 normal ovary or fallopian tube tissues) and immunohistochemistry by tissue microarrays (128 carcinomas and 112 normal ovary or fallopian tube tissues, benign or borderline ovarian tumor tissues) were performed to characterize expression of MAGE-A9 in EOC. We found that significantly higher MAGE-A9 mRNA expression in EOC tumors than that in normal ovary or fallopian tube tissues (all *P* < 0.05). Protein expression of MAGE-A9 was significantly associated with FIGO stage, high histological grade, level of CA-125 and metastasis. Consistent with the associated poor clinicopathologic features, patients with MAGE-A9^H (high-expressing)^ tumors had a worse overall survival as compared to patients with MAGE-A9^L (low or none-expressing)^ tumors. Further studies revealed that MAGE-A9 overexpression was an independent prognostic factor for overall survival (OS). Multivariate analysis showed that patients with MAGE-A9 overexpressing tumors had extremely poor OS. These findings indicate that MAGE-A9 expression may be helpful in predicting EOC prognosis.

Ovarian cancer, as one of the most common cancers worldwide, is the most lethal of all gynecologic malignant tumors with no less than 204,000 new cases and 125,000 deaths each year^1^. 5-year survival remains below 50% and tumor recurrence is the main factor for failure of ovarian cancer therapy following surgery^2^. Survival is much greater for women diagnosed with early ovarian cancer. With the development of a screening test, detecting early malignancy is seen as a priority by researchers^3^. If the tumor has spread by the time of diagnosis, surgery may be unobtainable. However, primary cytoreductive surgery and cytoreductive intervention are now accepted practice, and then subsequent treatment is usually chemotherapy^4^. Chemotherapeutic agents, such as alkylating agents, cytostatic antibiotics, platinum compounds, taxanes and topoisomerase modifiers, have been shown to be effective in EOC. However, so many agents take part in the management of EOC is an indication that none are entirely efficacious or appropriate in all circumstances^5^. Therefore, novel biomarkers with high sensitivity and specificity are urgently required for better diagnostic tools and targeted therapies of EOC^6^. Furthermore, novel biomarkers signify the era of personalized medicine has come to the real-world practice in cancer field. Not only have biomarkers contributed greatly to early detection, they have also significantly improved therapeutic effects^7^,^8^,^9^.

Cancer testis genes encode potential oncogenes that are activated in many human cancers of different histological types. Cancer testis antigens (CTAs), exclusively expressed in cancers, are candidate targets for anticancer immunotherapy and elicit cellular and humoral immune responses^10^. Melanoma-associated antigen (MAGE) genes which are classified as type 1 (MAGE-A, MAGE-B and MAGE-C) and type 2 (MAGE-D, MAGEE, MAGEF, MAGEH, MAGEL and NDN) and are almost all universally expressed are the best characterized members of the CTA family. Differences between MAGE genes are based on tissue-specific expression patterns and gene structures^11^,^12^. For example, malignancies of a broad range have been detected the expression of type 1 MAGE^13^,^14^,^15^,^16^,^17^.

MAGE-A is a multigene family consisting of 12 homologous genes (MAGE-A1–MAGE-A12) located on chromosome Xq28^18^,^19^ where they code for antigens that are recognized by cytolytic T lymphocytes (CTL). The promoters and the first exons of MAGE-A genes show considerable variability, indicating that regulation of MAGE-A family enables the same function expressed under different transcriptional controls. MAGE-A9 is one of the more frequently expressed CTAs in human tumors, including melanomas, head and neck squamous cell carcinomas, non-small-cell lung carcinomas, multiple myelomas, and hepatocellular carcinomas. Increasing evidence suggests that a contribution of MAGE-A genes family to cancer progression and metastasis and a relationship with a poor clinical outcome^20^,^21^. Based on these findings, the use of MAGE-A gene-based cancer immunotherapy is under clinical demonstration in several malignancies including metastatic melanoma and non-small cell lung cancer^22^,^23^.

As far as we know, MAGE-A9 protein expression in EOC and its correlation with clinical parameters have not yet been evaluated. Thus, we examined the expression of MAGE-A9 mRNA in fresh ovarian epithelial cancer tissue via the method of real-time PCR. Subsequently, we determined MAGE-A9 protein expression in ovarian epithelial tumor samples and analyzed the correlation between MAGE-A9 and other clinicopathologic features in a group of patients with EOC.

## Materials and Methods

### Clinical data and tissue samples

We selected patients who visited the gynecology department of the Affiliated Hospital of Nantong University, China between January 2005 and December 2009. Tissue samples were obtained at the time of surgery. Written informed consent was obtained from the patients for publication of this study and any accompanying images. There were 24 cases of normal ovarian tissues and 24 cases of normal fallopian tube tissues, 32 cases of ovarian benign tumor samples and 32 cases of borderline ovarian tumor samples, and 128 cases of ECO samples. Normal ovary and fallopian tube tissue samples from hysterectomy specimens resected for non-ovarian disease were collected for control. All ECO patients underwent standard surgery aiming for maximal tumor resection, including hysterectomy, bilateral salpingo-oophorectomy, pelvic and/or para-aortic lymphadenectomy and omentectomy. After resection, platinum-based chemotherapy was administrated for at least six cycles. None of the patients received chemotherapy, radiotherapy, or immunotherapy prior to surgery. Tissue specimens obtained were confirmed by histopathological examination and stored in liquid nitrogen 10 min after surgery. Patient clinical data were recorded in detail and the diagnoses were confirmed by at least two pathologists. Tumor histological grades and clinical stages were evaluated according to the pathological results after surgery. Clinical stages of ovarian cancer were based on FIGO (International Federation of Gynecology and Obstetrics) (presented in 2000) staging criteria. Of the 128 cases of ovarian cancer, there were 100 cases of serous carcinoma, 13 of endometrial carcinoma, and 15 of other types (5 cases of clear cell carcinoma, 4 cases of mucinous carcinoma, 4 cases of transitional cell carcinoma, and 2 cases of adeno-squamous carcinoma). There were 74 stage I-II, and 54 stage III -IV cases. With regard to histological grading, 99 cases were high grade and 29 were low grade. Patients were aged between 24 and 78 years, with an average age of 52.85 ± 15.66 years. Follow-up information on the study patients was updated through July 31, 2014 by reviewing medical records and data in the Chinese Public Security Bureau. Besides, another 12 carcinoma samples of high FIGO stage (stage III–IV), 12 carcinoma samples of low FIGO stage (stage I–II) and 20 normal ovary or fallopian tube tissue samples were collected for real-time PCR analysis. Study protocol was approved by the Ethics Committee of the Affiliated Hospital of Nantong University and all experiments were performed in accordance with approved guidelines of the Affiliated Hospital of Nantong University.

### RNA Isolation and Quantification of Transcript Levels

The total RNA was isolated according to the protocol of TRIZOL reagent (Life Technologies). Immediately after isolation, RNA quantity and quality was determined by the method of spectrophotometry. Complementary DNA was then synthesized from 1 μg of total RNA with reverse transcription Kit (Fermentas) according to the manufacturer’s instructions. The mRNA expressions of MAGE-A9 and β-actin were measured by real-time PCR system (Applied Biosystems, Carlsbad, USA). The data were obtained by normalizing MAGE-A9 gene Ct (cycle threshold) values with corresponding β-actin Ct, and then analyzed with 2-ΔΔCt Ctmethod. The primers sequences are as follows: MAGE-A9 forward primer (5’ -CAC TGT ATG TCA TCT CTG -3’) and MAGE-A9 reverse primer (5’-ACT ACT GTC ATT CAT TAA CT -3’), β-actin forward primer (5’-GGC GGA CTA TGA CTT AGT TG -3’) and β-actin reverse primer (5’-AAA CAA CAA TGT GCA ATC AA -3’).

### Immunohistochemical staining and evaluation

We used tissue microarray system (Quick-Ray, UT06, UNITMA, Korea) in the Department of Clinical Pathology, Nantong University Hospital, Jiangsu, China. Core tissue biopsies (2 mm in diameter) were taken from individual paraffin-embedded sections and arranged in recipient paraffin blocks. TMA specimens were cut into 4-μm sections and placed on super frost-charged glass microscope slides.TMA analysis was used as a quality control for hematoxylin and eosin staining. Tissue sections were deparaffinized and rehydrated in graded ethanol. Antigen retrieval was performed by boiling sections in ethylenediaminetetra-acetic acid buffer, pH 6.0, for 3 min in a pressure cooker. Endogenous peroxidase activity was quenched with 3% hydrogen peroxide for 30 min. Sections were then incubated with a monoclonal antibody specific to MAGE-A9 (dilution 1:50) (Abcam, ab66904) at 4 °C overnight, followed by incubation with a biotinylated anti-rabbit secondary antibody at 37 °C for 30 min. Slides were then processed using horseradish peroxidase and 3, 3-diaminobenzidine chromogen solution and counterstained with hematoxylin. The staining intensity of MAGE-A9 for each slide was evaluated and scored by two independent pathologists. Staining intensity was scored as follows: 0 (negative), 1(weakly positive), 2 (moderately positive), and 3 (strongly positive).The percentage of positive cells was scored as follows: 0 for 0–20%, 1 for 21–50%, 2 for 51–75%, and 3 for 76–100%. The product of the percentage and intensity score was used as the final staining score, as described previously^24^.

The cutoff point for the MAGE-A9 expression score that was statistically significant in terms of overall survival was set using the X-tile software program (The Rimm Lab at Yale University; http://www.tissuearray.org/rimmlab) as described previously^25^. The cutoff 140 was selected to evaluate: score 0-140 was considered low expression while 141–300 was considered high expression. For all subsequent analyses, MAGE-A9 protein expression levels were considered either as “Low” or “High” using these cutoff values^24^.

### Statistical analysis

Statistical calculations of the PCR data were performed using t test when two groups are compared. One-way ANOVA followed by Tukey multiple comparison test was used when three or more groups were compared. χ^2^ tests were performed to evaluate whether MAGE-A9 expression was correlated with clinicopathologic parameters. For TMA slides, age and other clinicopathologic information were evaluated. Patient outcome survival curves were calculated using the Kaplan–Meier method. Factors shown to be of prognostic significance in univariate models were evaluated in a multivariate Cox regression model. For all analyses, a *P*-value < 0.05 was regarded as statistically significant. Data were analyzed using SPSS 20 statistics software (SPSS Inc., Chicago, IL, USA) and STATA 12.0 (StataCorp, College Station, TX, USA).

## Results

### MAGE-A9 mRNA expression in EOC patients by PCR

**Figure 1:**
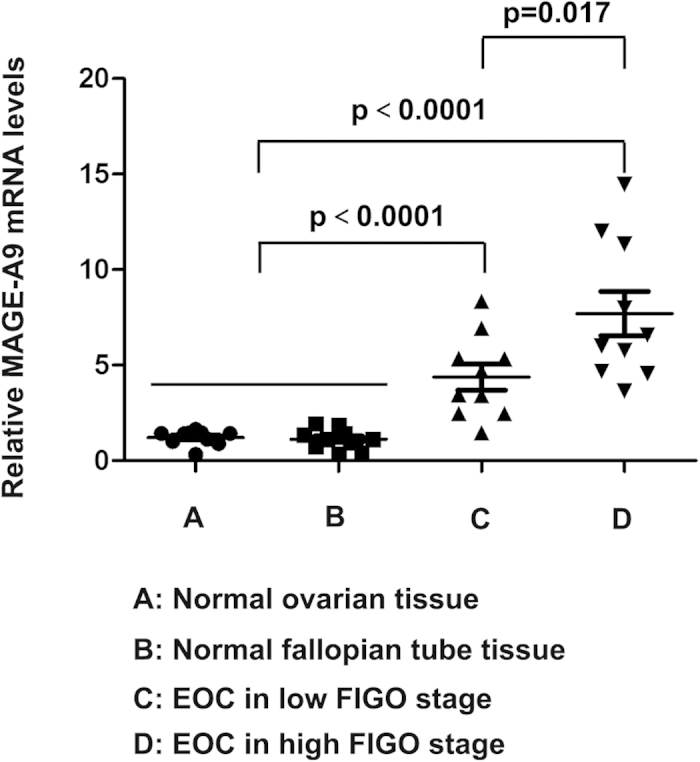
MAGE-A9 mRNA expression in EOC tissues and normal tissues. Real-time PCR demonstrated that the expression of MAGE-A9 mRNA in high FIGO stage, low FIGO stage, normal ovary and normal fallopian tube tissue were 7.63 ± 0.503, 4.13 ± 0.284, 1.03 ± 0.128 and 1.475 ± 0.093 respectively. The expression of MAGE-A9 mRNA in ovarian cancer samples was significantly higher than in noncancerous tissues (all *P* < 0.05).

To evaluate the MAGE-A9 mRNA expression in EOC patients, RNA was isolated from 12 high FIGO stage (III–IV) and 12 low FIGO stage (I–II) using real-time PCR. When comparing the expression in noncancerous tissues, 10 normal ovary and 10 normal fallopian tube tissue samples were collected. As is shown in Fig. 1, the means of MAGE-A9 mRNA in stage III–IV, stage I–II and normal ovary and normal fallopian tube tissue were 7.63 ± 0.503, 4.13 ± 0.284, 1.03 ± 0.128 and 1.475 ± 0.093 respectively. The expression of MAGE-A9 mRNA in ovarian cancer samples was significantly higher than in noncancerous tissues (all *P* < 0.05).

### MAGE-A9 protein expression patterns in tissue arrays of EOC patients by HIC

**Figure 2:**
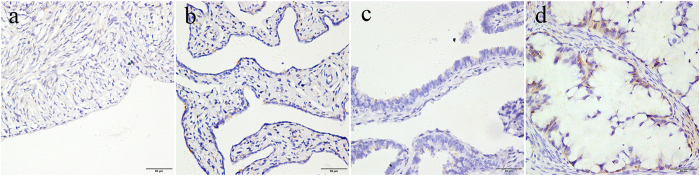
Immunohistochemical (IHC) staining for MAGE-A9 expression in normal ovarian tissue, normal fallopian tube tissue, a benign tumor and a borderline ovarian tumor sample. **a** Negative IHC staining of MAGE-A9 in normal ovarian tissue; **b** Negative IHC staining of MAGE-A9 in normal fallopian tube tissue; **c** Negative IHC staining of MAGE-A9 in a benign ovarian tumor; **d** weak MAGE-A9 staining in a borderline ovarian tumor sample. Original magnification ×400 (scale bars 50 μm).

TMA-based immunohistochemistry studies were carried out to confirm MAGE-A9 expression in EOC patients at the tissue level. Results showed that MAGE-A9 expression was significantly upregulated in EOC patients but negative or low in normal ovarian tissue, normal fallopian tube tissue, benign tumor, and borderline ovarian tumor samples (Fig. 2). Positive staining was predominantly localized in the cytoplasm of EOC cells. High cytoplasmic expression of MAGE-A9 was observed in 36.72% (47/128) of EOC tumors compared with only 6.25% (2/32) of benign tumors and 3.13% (1/32) of borderline ovarian tumors. The data showed statistical significance using χ^2^ test analysis (χ^2^ = 42.426, *P* < 0.001) (Table 1).

### Association of MAGE-A9 expression with clinicopathologic parameters

**Figure 3:**
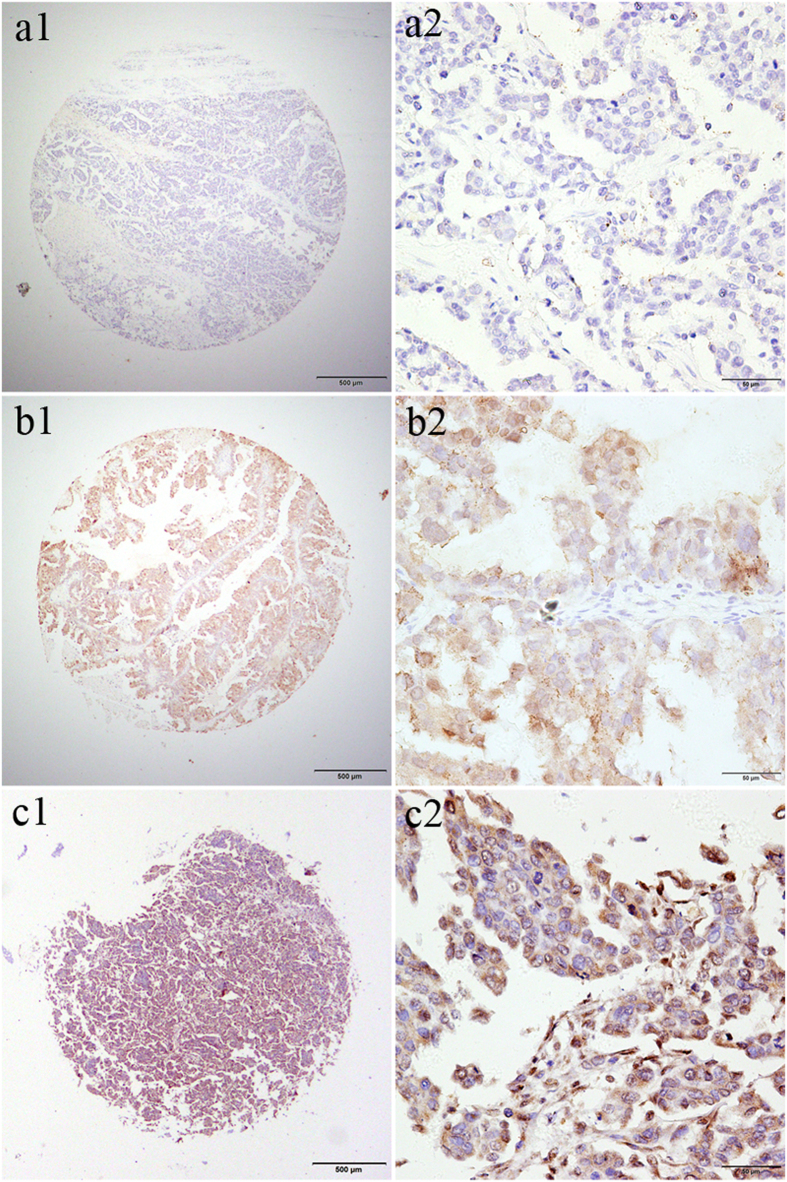
Expression of MAGE-A9 in EOC with tissue microarray (TMA). samples immunostained for MAGE-A9 showed cytoplasmic positivity. MAGE-A9 protein expression in tumors from EOC patients showed three different levels. a1 and a2 showed negative IHC staining of MAGE-A9, b1 and b2 showed weak IHC staining in EOC samples. While c1 and c2 showed strong IHC staining of MAGE-A9 in EOC samples with advanced cancer, expressing high MAGE-A9 levels. Original magnification ×40 in a1, b1, c1 (scale bars 500 μm); ×400 in a2, b2, c2 (scale bars 50 μm).

Associations of MAGE-A9 expression and clinicopathologic factors are summarized in Table 2. Stratifying clinical characteristics by the two MAGE-A9 expression groups, we observed that MAGE-A9 protein positivity was significantly associated with FIGO stage (*P* = 0.001), tumor grade (*P* = 0.042), level of CA-125 (*P* = 0.026) and metastasis (*P* = 0.014) (Table 2). We found no significant association between MAGE-A9 expression and patient age, histological type, or ascites cells in our study (Table 2). Expression of MAGE-A9 in ovarian cancer was extremely high but negative or low in normal ovarian tissue, normal fallopian tube tissue, benign tumor, and borderline ovarian tumor samples. Moreover, frequently expressed MAGE-A9 was especially related to FIGO stage, tumor grade and metastasis (Fig. 3). This phenomenon may be associated with advanced cancer, which was indicated by tumor grade, CA-125 level, metastasis, and FIGO stage.

### Overexpression of MAGE-A9 predicts poor prognosis

**Figure 4:**
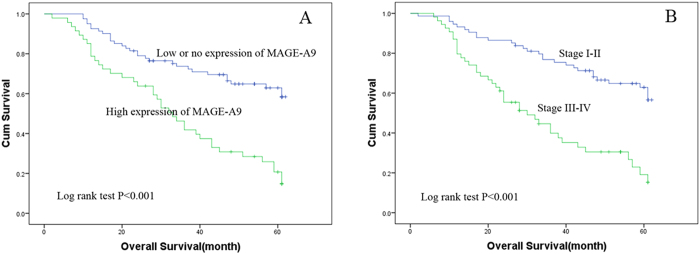
Kaplan–Meier plots using the log rank survival test. **A** Overall survival rate in patients with high MAGE-A9 expression was significantly lower than that in patients with low and no MAGE-A9 expression. (*P* < 0.001). **B** Overall survival rate in patients with high FIGO stage was significantly lower than that in patients with low FIGO stage. (*P* < 0.001).

Multivariate analysis was performed using the Cox proportional hazards model for all of the significant variables in the univariate analysis. In the univariate survival analysis, expression of high level of MAGE-A9 expression (HR 2.944; *P* < 0.001), ascites cell (HR 1.850; *P* < 0.001), metastasis (HR 3.778; *P* < 0.001) and FIGO stage (HR 1.772; *P* < 0.001) were associated with OS (Table 3). Factors shown to be of prognostic significance in univariate models were evaluated in a multivariate Cox regression model. FIGO stage already included ascites cell and metastasis, so we removed the latter two factors in the multivariate analysis. In the multivariate Cox regression model, the results demonstrated that MAGE-A9 overexpression (HR 2.271; *P* < 0.001) and FIGO stage (HR 1.569; *P* < 0.001) were unfavorable prognostic factors independent of other clinicopathological factors (Table 3). Patients with MAGE-A9^H^ had a poor OS compared to patients with MAGE-A9^L^ tumors, as well as advanced FIGO stage compared to low FIGO stage, as shown in the Kaplan–Meier plot (log rank, *P* < 0.001; Fig. 4).

## Discussion

Immunotherapy is an attractive approach to improve therapeutic effects and survival rates in EOC. The MAGE families CTA, possible tumor antigen targets, are regarded as the most promising candidates of anti-cancer vaccines. Sayeema *et al.* found that MAGE-A1, MAGE-A4, MAGE-A3, and MAGE-A10 are promising candidate targets for cancer immunotherapy in EOC patients^26^. To the best of our knowledge, there have been no previously reported studies examining the potential effect of MAGE-A9 on the survival of patients with EOC. In our study, we found that MAGE-A9 protein expression was significantly linked to advanced cancer, indicated by tumor grade, CA-125 level, metastasis, and FIGO stage. These strong associations suggest that MAGE-A9 could be potentially used as a novel biomarker of a more aggressive phenotype of EOC, promoting tumor invasion and metastasis. So, better insights into the function of MAGE-A9 gene, which could be the targets of antitumor therapies, may shed light on the link between EOC and tumor development.

MAGE-A9 is frequently expressed in a variety of cancers^27^,^28^,^29^,^30^,^31^,^32^,^33^,^34^,^35^, which can provide additional prognostic information in renal cell carcinoma, bladder cancer, hepatocellular carcinoma, laryngeal squamous cell carcinoma, cutaneous T cell lymphomas and breast cancer. Although the physiological function of MAGE-A proteins are still poorly understood, increasing evidence suggests that they are involved in the initiation of cancer formation, including the regulation of cell cycle progression and cell apoptosis^36^. MAGE-A proteins expression are also unregulated in chemotherapy (paclitaxel) resistant ovarian cancer, melanoma, and multiple myeloma cell lines, compared with chemotherapy susceptible varieties^37^. Therefore, it is possible that MAGE-A9 expression favors tumor cell survival and that MAGE-A9 proteins function as oncoproteins. Previous studies revealed an inverse relationship between dendritic cells (DCs) and MAGE-A expression, which may indicate that MAGE-A-positive tumor cells would be akin to tumor stem cells by escaping the host immune response and promote cancer prognosis^38^. MAGE-A9 mRNA expressions in ovarian cancer tissues were higher than those in normal ovary and normal fallopian tube tissues according to our real-time PCR result. MAGE-A9 protein expression in tissue arrays of EOC patients by HIC also revealed that over expression in ovarian cancer tissues. This result is similar to the previous studies of different malignancies that indicated frequent expression of MAGE-A9 in cancer tissues^27^,^28^,^29^,^30^,^31^,^32^. The results of our present study showed that positive MAGE-A9 staining was significantly related to FIGO stage. Overexpression of MAGE-A9 protein was associated with an increased risk of metastasis and was significantly related to poor survival outcomes. These clinical findings suggest that increased MAGE-A9 expression correlates with invasive behavior and metastatic processes of EOC. Multivariate analysis showed that increased MAGE-A9 expression and advanced FIGO stage independently predicted unfavorable overall survival of ovarian cancer patients. Although limitations include the small number of patients with relatively short follow-up time, our results first reported that MAGE-A9 could be used as a novel biomarker for improving clinical outcomes of EOC patients after surgery.

In summary, this study has provided critical insight into the role of MAGE-A9 in the progression of EOC. The frequent upregulation of MAGE-A9 expression in human EOC highlights its potential as a novel therapeutic target for EOC. The findings reported here also indicate that MAGE-A9 overexpression was associated with a poor survival rate, which might be helpful in designing future studies aimed at understanding the molecular development of EOC. The potential clinical value of MAGE-A9 as a novel biomarker in EOC should be investigated in randomized controlled studies.

## Additional Information

**How to cite this article**: Xu, Y. *et al.* Overexpression of MAGE-A9 Is Predictive of Poor Prognosis in Epithelial Ovarian Cancer. *Sci. Rep.* **5**, 12104; doi: 10.1038/srep12104 (2015).

## Figures and Tables

**Table 1:** MAGE-A9 immunohistochemical staining of protein in normal ovarian, normal fallopian tube, benign ovarian tumor, borderline ovarian tumor and EOC tissues.

Tissue sample		MAGE-A9 expression			
	n	Low or none	High	Pearson χ2	*P*- value
Normal ovarian tissue	24	24(100.00)	0(0.00)	42.426	0.000*
Normal fallopian tube tissue	24	24(100.00)	0(0.00)		
Benign ovarian tumor	32	30(93.75)	2(6.25)		
Borderline ovarian tumor	32	31(96.88)	1(3.13)		
EOC	128	81(63.28)	47(36.72)		

**Table 2:** Patient clinicopathologic characteristics according to MAGE-A9 scores.

Groups	MAGEA9				
	n = 128	Low or no (n = 81)	High (n = 47)	Pearson χ2	*P-*value
Age at diagnosis				0.693	0.405
≤60 years	84	51(60.71)	33(39.29)		
>60 years	44	30(68.18)	14(31.82)		
FIGO stage				11.597	0.001*
1 ~ 2	74	56(75.68)	18(24.32)		
3 ~ 4	54	25(46.30)	29(53.70)		
Histological classification				4.226	0.121
serous carcinoma	100	65(65.00)	35(35.00)		
endometrioid carcinoma	13	5(38.46)	8(61.54)		
other^a^	15	11(73.33)	4(26.67)		
Grade				4.146	0.042*
Low	29	23(79.31)	6(20.69)		
High	99	58(58.59)	41(41.41)		
Ascites cell				0.068	0.795
No	70	42(60.00)	28(40.00)		
Yes	28	16(57.14)	12(42.86)		
Unknown	30	21	9		
Serum CA-125 (U/ml)				4.969	0.026*
≤100	12	12(100.00)	0(0.00)		
>100	89	62(69.66)	27(30.34)		
Unknown	27	8	19		
Metastasis				6.096	0.014*
No	70	51(72.86)	19(27.14)		
Yes	58	30(51.72)	28(48.28)		

*P < 0.05 indicates a significant association among the variables; Metastasis: pelvic lymph node metastases or nearby tissues and organs involved.

a, others: clear cell carcinoma, 5 cases; mucinous carcinoma, 4 cases; transitional cell carcinoma, 4 cases; adeno-squamous carcinoma, 2 cases.

**Table 3:** Univariate and multivariate Cox proportional hazard models of overall survival.

Variable	Univariate analysis			Multivariate analysis		
	HR	*P-*value	95% CI	HR	*P-*value	95% CI
MAGE-A9						
Low vs. High	2.944	0.000*	1.820–4.763	2.271	0.001*	1.372–3.761
Age (years)						
<60 vs. ≥60	1.484	0.112	0.912–2.415			
FIGO Stage						
I -II vs. III- IV	1.772	0.000*	1.386–2.265	1.569	0.001*	1.213–2.030
Histological type						
Sc vs. Ec vs. Others	0.982	0.922	0.681–1.415			
Grade						
Low vs. High	1.880	0.056	0.984–3.591			
Ascites cell						
Yes vs. No	1.850	0.025*	1.080–3.171			
Serum CA-125 (U/ml)						
<100 vs. ≥100	2.522	0.120	0.785–8.106			
Metastasis						
Yes vs. No	3.778	0.000*	2.277–6.268			

Sc, serous carcinoma; Ec, endometrioid carcinoma; HR: Hazard ratio; CI: Confidence interval.

^*^P < 0.05.
